# Qualitative evaluation: A critical and interpretative complementary approach to improve health programs and services

**DOI:** 10.3402/qhw.v9.24417

**Published:** 2014-08-22

**Authors:** Luz María Tejada Tayabas, Teresita Castillo León, JOEL MONARREZ ESPINO

**Affiliations:** 1 Faculty of Nursing, San Luis Potosi Autonomous University, San Luis Potosi, Mexico; 2 Faculty of Psychology, Autonomous University of Yucatan, Yucatan, Mexico; 3 Department of Public Health Sciences, Karolinska Institutet, Stockholm, Sweden

**Keywords:** Qualitative evaluation, qualitative research, health programs, health care services

## Abstract

This short essay aims at commenting on the origin, development, rationale, and main characteristics of qualitative evaluation (QE), emphasizing the value of this methodological tool to evaluate health programs and services. During the past decades, different approaches have come to light proposing complementary alternatives to appraise the performance of public health programs, mainly focusing on the implementation process involved rather than on measuring the impact of such actions. QE is an alternative tool that can be used to illustrate and understand the process faced when executing health programs. It can also lead to useful suggestions to modify its implementation from the stakeholders’ perspectives, as it uses a qualitative approach that considers participants as reflective subjects, generators of meanings. This implies that beneficiaries become involved in an active manner in the evaluated phenomena with the aim of improving the health programs or services that they receive. With this work we want to encourage evaluators in the field of public health to consider the use of QE as a complementary tool for program evaluation to be able to identify areas of opportunity to improve programs’ implementation processes from the perspective of intended beneficiaries.

## Introduction

In most countries, the way in which most health programs and services have been assessed has usually followed a positivist paradigm. This type of assessment includes economic evaluations to measure the cost-benefit of a specific program, epidemiological evaluations to determine the efficacy or effectiveness of an intervention, and managerial evaluations to measure indicators and the accomplishment of specific goals.

During the past decades, different approaches have come to light proposing alternatives to appraise the performance of public health programs, mainly focusing at the implementation process involved rather than at measuring the impact of such actions (Bosi & Mercado, 2006). Qualitative evaluation (QE) is one of these alternative methods to assess health programs and services. Yet, QE are still scarce due to the poor knowledge and understanding of their potential to complement more conventional evaluations; this has resulted in relatively few articles being published, which in turn limits the promotion of this approach.

Presenting a comprehensive review of contributions to the field of QE is a difficult task, and indeed not the purpose of this work. Instead, we evoke some representative authors that have used this approach to improve health systems serving vulnerable and marginalized groups. Among these, Patton (2002) has theorized extensively about when, where, and how qualitative approaches can be used to assess health programs.

Evaluations known as fourth-generation (Guba & Lincoln, 1989), democratic (Simons, 1999), empowering (Fetterman, 2001), participative (Springett, 2002), critical (Everitt & Hardiker, 2003), and qualitative (Shaw, 2003) have been some of the terms given to designate approaches that use an interpretative, critical, and participative paradigm (Greene, 2000) to assess the functioning of social and health initiatives. Among other goals, these methods seek to improve the management of public programs by promoting the democratization of institutions, by strengthening the transparency of the processes involved and empowering the programs’ beneficiaries to take a more active role, and by promoting the participation of individuals or groups traditionally excluded (Mercado, Tejada-Tayabas, & Springett, 2008).

In particular, the QE approach has tried to expose practical issues that affect programs or policies in real settings, using the experiences and perceptions of the stakeholders. The method, grounded in the epistemological perspective and traditions of qualitative research (e.g., ethnography, phenomenology, ethnomethodology, and grounded theory) aims at improving the understanding and interpretation of phenomena from the standpoint of the participants involved. For this reason, QE requires direct contact with the socioeconomic and cultural environment where the actions assessed are taking place to understand and interpret the findings correctly (Shaw, 2003).

In this context, this assay aims at commenting on the origin, development, rationale, and characteristics of QE, highlighting the value of this approach to evaluate health programs and services.

## Origin and development

Theoretical contributions made by specialists in the field of evaluative research over the last decades were characterized by the dissemination of different evaluative models that exhibited the authors’ stance defining what the evaluative process was and how it should be conducted. This was a period of prolific conceptual and methodological plurality. At the same time, those using traditional evaluation methods became hesitant about the validity of the knowledge being produced leading to a diversification of the evaluation uses and purposes. Some authors simplified such diversity of evaluation models into quantitative and qualitative (Guba & Lincoln, 1989). In fact, this dichotomization still remains common in settings where there is a limited practice to use mixed designs to approach health topics (Mercado, Diaz, Tejada-Tayabas, Ascencio, 2011).

Chronologically, QE was initially used in the 70s when assessing and analyzing public policies to understand their fundamentals, direction, purpose, and implementation (Spencer, Ritchie, Lewis, & Dillon, 2003). Thereafter, the development of QE followed two phases. The first occurred in the post-positivistic atmosphere of the 80s, whereby a growing number of authors tried to incorporate qualitative procedures into mainstream quantitative evaluation practices. Open-minded researchers from North America and Europe started employing these methods to support quantitative findings using a different pathway to approach reality. This combination of methodological tools and approaches proved useful to better understand and interpret phenomena under real settings and circumstances, and highlighted the potential of qualitative research in the search for solutions of social problems (Campbell, 1978).

The second phase occurred when the interpretative paradigm was formalized in the 90s (Greene, 2000); then the focus shifted to the audience. The role of the participants in evaluated programs became a priority, setting the basis for a transactional and phenomenological relationship between the evaluator and the audience. Here, the programs’ stakeholders (i.e., patients, program users, health providers, and administrative personnel involved) take a leading part in the evaluation and decision-making process (Lincoln & Guba, 1986). Reality was conceived and conceptualized qualitatively, and critical analyses of subjective data provided clues about how intricate processes worked. A wide range of subjects’ actions, structures, perceptions, and relations between micro and macro realities were expressed in the evaluated phenomenon (Minayo & Neto, 1999).

Accordingly, QE combined theoretical, ideological, and methodological elements to better understand stakeholders’ perspectives about programs with the ultimate goal of implementing transformative actions by integrating research and participative tasks to generate changes in intervention contexts. The participative evaluation (Garaway, 1995; Papineau & Kiely, 1996; Smith, 1999) and participative action research models (Cassell & Johnson, 2006) were some of the approaches that occurred during this second phase of the QE development.

One scheme that tried to combine qualitative and participative methodologies to generate transformative action was the fourth generation model (Guba & Lincoln, 1989). This relativistic and constructivist model opposed the traditional evaluation model by assuming the existence of multiple realities socially constructed, and supported the use of a contextual, holistic, and intersubjective inquiry. Emphasis was given to the participation of the stakeholders involved, not only to question, interpret, and understand their perception, but also to promote actions for change. By proposing this model, the authors attempted to shift the traditional top-down approach to a more horizontal application, giving the programs’ participants a protagonist role in the evaluation process. They also established a circular methodology by which findings could be analyzed, reflected upon, and reconstructed by the participants themselves. These elements eventually became the foundation of the QE model with its interpretative, comprehensive, and constructivist character.

Later, Weiss (1990) reflected and analyzed the political context in which evaluations occurred, and questioned the traditional model by pointing out its tendency to satisfy the institutional establishment, giving little margin for interference or change in the course of the programs.

In the area of participatory action, Chambers (1994) proposed a rural appraisal, used in various low-income countries from Latin America, Africa, and Asia to integrate the opinions and knowledge of rural inhabitants when planning and implementing development projects; some NGOs even adopted this approach to follow-up and assess reproductive, environmental, food safety, and nutritional programs.

Already in the mid-90s, the receptive evaluation model proposed by Stake (1995) underscored the importance of qualitative inquiry and participative methods more in line with the program's activities than with its objectives, giving relevance to the values expressed by stakeholders of the evaluated programs. Therefore, the contribution of this model became methodological, as it aimed at describing the programs rather than to transform them.

In the past decade, House and Howe (2001) developed the notion of reaching social justice through evaluation means. They incorporated QE to reveal details of the programs, conceived as irregular and changeable entities. The participants’ perceptions were considered essential, and emphasis was given to the principle of equity.

## Rationale and central features

QE is characterized by an emerging construction of theoretical elements, methods, techniques, and instruments that are incorporated into the evaluation puzzle to understand and change program practices. It is therefore an activity that involves a constant process of reflection, introspection, and decision making. It has a naturalistic character, as it allows studying activities and events as they occur in reality. It is open and sensible to depict processes, but mostly events, concepts, needs, meanings, expectations, feelings, challenges, and problems experienced daily by the stakeholders involved. It is thus particularly useful to study programs’ variations in the process of implementation from one place to another, and from the perspective of different individuals (Kerber, Kirchhof, Cezar-Vaz, & Silveira, 2010). QE can capture the nature of these variations, and the modifications and contradictions that occur when programs are executed given the distinct idiosyncrasy of the participants and the specificity of each experience. Such issues can hardly be measured or predicted beforehand. The most relevant features of QE according to Patton (2002), a representative author, are displayed in Table I.

**Table I:** Main characteristics of the qualitative evaluation

– It is useful to emphasize the analysis of the individual perspective of the participants, and more so when actors are affected differently, making it necessary to describe, analyze, interpret, and compare these various perspectives
– It can reveal and clarify the internal dynamic of a program; its strengths and weaknesses, making evident those details related with its operation as expressed by the participants
– Seeks to explore and discover phenomena using an inductive logic centered on the actors’ perspectives and the specific context in which programs are implemented
– It implies direct and personal contact with the people involved in the program
– Tries to understand the perspective of others; it is essential to exercise empathy and systematic introspection that can only be gained through interpersonal communication
– It shares many characteristics of the qualitative research, such as the interest to assess the qualities of social events, and to disclose their heterogeneity and relational logic
– It is grounded in alternative paradigms critical to positivistic approaches, arguing for different ways to conceive reality and to generate knowledge (epistemological element)
– It is flexible with the design and use of methods and techniques to approach social processes in a natural way (methodological element)
– It supports the need to reflect on the importance of knowing the social context (theoretical element)
– Takes a stand concerning the human problems being investigated (ideological element)
– This latter ideological component exposes, questions, criticizes, and condemns the *status quo* and the conservative practices of conventional evaluation, which has failed to solve many of the problems in its social and cultural context

*Source*: Patton (1990).

The political side of the QE relies on the fact that the results produced can influence the political agenda. It dilutes the dominant perspective that confers superiority to the technical and scientific vision over the critical and interpretative approach by giving relevance to other different perceptions, meanings, experiences, and practices experienced by programs’ participants. On the other hand, it exposes power relationships among various stakeholders who are social subjects that do not remain passive, and who exert actions that can affect the program's development generating potential changes (Potvin, Gendron, & Bilodeau, 2006). As a result, conducting QE becomes a relevant process of social intervention. However, for this to occur, it is essential to transform the knowledge acquired into practical action, namely, to make of the evaluation results tools to design and implement interventions that promote change.

## Value of this approach

The debate of whether evaluation is a different activity than research has been undertaken by several authors (e.g., House, 1980; Lincoln & Guba, 1986; Patton, 2002; Scriven, 1986). Fournier (1995) and Shaw (2003) tried to specifically differentiate between these two. Shaw (2003) mentioned that evaluation seeks to address practical problems and short-term issues, and calls for action, in contrast with research that addresses theoretical topics, strives for description, and looks at long-term issues. Hammersley (2002, 2003) tried to summarize the difference by stating that while the first refers to practical research, the second deals with scientific research. In any case, it is not possible to conduct a meaningful evaluation without using research methods. Therefore, perhaps the most important distinction lies in the purpose and products rather than in the methodological differences.

In contrast with qualitative research, QE deals with practical issues, with the potential of promoting change in the course of programs, and leads to judgments of merit and value linked to a specific context and population. Yet, QE faces the same criticisms regarding trustworthiness, reliability, and functionality that affect any qualitative investigation. In this context, subjectivity remains as an important concern, especially by those using conventional quantitative evaluation methods.

So far, there are signals suggesting that QE will become more widely used in the coming years as an alternative or complementary approach to evaluate health programs. This implies the recognition that this approach can use findings effectively to illustrate and reflect upon difficulties in the implementation of programs with the ultimate goal of improving the processes involved from the perspective of the stakeholders.

We believe that rather than arguing for or against the relative advantages or disadvantages among the various evaluative approaches, attention should be given to those capable of generating relevant information that can be useful to improve programs’ aims, always keeping in mind the importance of the opinions, perceptions, and expectations of the individuals that programs are supposed to serve, as well as those of other stakeholders involved in the design, planning, and execution of programs.

The participation of social actors in the assessment of programs and the complexity of the evaluated phenomena are key elements that constrain the possibility of quantitative methods alone to appreciate a reality filled with inter-subjectivity, experiences, meanings, and interpretations, as several reserachers have tried to illustrate in several studies.

As to the range of programs that can be assessed using QE, the list of examples is broad. While some authors have qualitatively assessed specific health programs, such as those to detect, treat, and control patients with cancer (Collie et al., 2014; Tejada-Tayabas, Hernández-Ibarra, & Pastor-Durango, 2012), others have evaluated programs to prevent depression in adolescents (Iloabachie et al., 2011). QE has also been utilized to examine the availability and access to medical services (Tejada-Tayabas & Mercado, 2010), to value digital sources of information and support for patients with diabetes (Jeremy et al., 2011), to strengthen the evaluation process to assess the performance of nursing professionals (Gonçalves, Lima, Crisitano, & Hashimoto, 2007), or to assess patients’ satisfaction of hospital services (Paiva & Gomes, 2007).

In general, the results derived from these studies have provided useful information not only for designing and planning purposes, but to implement actions to improve the effectiveness of the programs. Even though they used a critical approach, they all proposed concrete strategies for the stakeholders based on perceptions, experiences, needs, and expectations, facilitating the decision-making process to plan and execute specific actions.

They have been able to gain insights about what is really happening in the programs: their strengths and weaknesses, their contradictions and conflicts, and the gap between the intended and experienced implementation. These attempts are noteworthy, as they challenge and encourage other researchers and evaluators to value the use of this approach to assist policy makers to improve existing health programs.

It is also important to keep in mind that diversity rather that uniformity is the norm when designing and carrying out QEs. Aims, methods, techniques, types, and potential use of the results, as well as the intended role of the evaluator can vary considerably, making it undesirable to judge one approach as better than the other. Presenting the framework of reference and assessing the proposed methods and techniques in context with the purpose of the evaluation is what actually determines the relevance of the specific approach used. Moreover, evaluative approaches can indeed be complementary. The fact that we generally evaluate complex and dynamic systems that often involve problematic individuals with intricate interactions inside and outside the system makes it suitable to combine approaches to better unveil such complexity.

## Final considerations

We would like to encourage academic evaluators in the field of public health to get more directly involved with the reality they investigate so that their efforts are more conducive to its transformation, as the very purpose of evaluating is to integrate knowledge and action to promote health improvements. We believe that researchers could learn to move comfortably from the dimension of investigating phenomena aiming at understanding and generating knowledge, to the dimension of transforming society, which in this context refers to taking an active role to promote changes that result in a better health for the population.

QE is then an alternative tool to illustrate and to understand the process faced when executing a health program, and proposes potential ways to modify its implementation from the participants’ perspectives using a qualitative approach that considers these actors as active and reflective subjects, generators of meanings. This implies that beneficiaries become involved in an active and pro-active manner in the evaluated phenomena with the aim of improving the very health programs or services they receive.

Qualitative and participative models can complement each other. Participative processes can hardly be effective to achieve changes if deep understanding of the stakeholders’ perspectives is missing, which is what QE provides. Although participative evaluation combines discovery and activism to transform society, this practice occurs depending on the way in which people live and conceive the world, which can be unveiled through qualitative and interpretative methods. Conversely, while QE provides understanding of the reality from the stakeholders’ perspective, it cannot alone generate the conditions for change to transform such reality as participative evaluation does.

This reflection points to the importance of considering quantitative, qualitative, and participative approaches as complementary rather than conflicting to be able to generate more complete, meaningful, and transformative evaluations.

The fact that there is a plurality of methods, techniques, results, effects, and topics of evaluation along with the various functions that the evaluation can take and the roles that the evaluator can play, make it clear that there is not a single model to be used, nor is it possible to typify approaches as better or worse. All this points to the importance of contextualizing the topic with the purpose of the evaluation to select a combination of methods that best achieve the goals of the evaluation. Such combinations are capable of assessing intricate and dynamic systems that involve individuals with complex interactions inside and outside the system in which they operate to effectively integrate knowledge and action (Potvin et al., 2006).
